# Integrative analyses shed new light on human ribosomal protein gene regulation

**DOI:** 10.1038/srep28619

**Published:** 2016-06-27

**Authors:** Xin Li, Yiyu Zheng, Haiyan Hu, Xiaoman Li

**Affiliations:** 1 Department of Electrical Engineering & Computer Science, University of Central Florida, Orlando, FL, 32816, USA; 2 Burnett School of Biomedical Science, University of Central Florida, Orlando, FL, 32816, USA

## Abstract

Ribosomal protein genes (RPGs) are important house-keeping genes that are well-known for their coordinated expression. Previous studies on RPGs are largely limited to their promoter regions. Recent high-throughput studies provide an unprecedented opportunity to study how human RPGs are transcriptionally modulated and how such transcriptional regulation may contribute to the coordinate gene expression in various tissues and cell types. By analyzing the DNase I hypersensitive sites under 349 experimental conditions, we predicted 217 RPG regulatory regions in the human genome. More than 86.6% of these computationally predicted regulatory regions were partially corroborated by independent experimental measurements. Motif analyses on these predicted regulatory regions identified 31 DNA motifs, including 57.1% of experimentally validated motifs in literature that regulate RPGs. Interestingly, we observed that the majority of the predicted motifs were shared by the predicted distal and proximal regulatory regions of the same RPGs, a likely general mechanism for enhancer-promoter interactions. We also found that RPGs may be differently regulated in different cells, indicating that condition-specific RPG regulatory regions still need to be discovered and investigated. Our study advances the understanding of how RPGs are coordinately modulated, which sheds light to the general principles of gene transcriptional regulation in mammals.

Ribosomal protein genes (RPGs) are important house-keeping genes that code for the architecture proteins in ribosomes, the machinery responsible for protein synthesis^1^. There are 80 RPGs scattered throughout the human genome, each of which is a single-copy gene^1^. These 80 genes are active under every condition in every human cell type studied so far^2^. Their mRNA expression levels are highly correlated across different experimental conditions, different cell types, and even different species^3^,^4^,^5^,^6^,^7^,^8^,^9^. For instance, in many gene expression studies in various species, RPGs always stand out as a group of highly correlated genes^10^,^11^. Although such a coordinated expression pattern of RPGs is well-known, our understanding of how such a pattern is orchestrated is still limited^12^.

To explain the coordinate expression pattern of RPGs, many studies hypothesized and suggested that RPGs in a species may be regulated by the same transcription factors (TFs) and share common motifs in their regulatory regions^13^,^14^,^15^,^16^,^17^. Regulatory regions are genomic regions of several hundred to several thousand base pairs (bp) long, where regulatory proteins bind to regulate the expression of their target genes. For instance, Wagner *et al.* identified a TF motif CTTCCYTYYTC in regulatory regions of all three mouse RPGs they tested^13^. Hariharan *et al.* observed the TF motif GCNGCCATC in the regulatory regions of all four mouse RPGs for which they did experiments^14^. A computational study identified shared TF motifs by 78 of the 80 human, mouse, and rat RPGs^15^. This study also identified common motifs in the majority of RPGs in yeast, plants, worms and insects^15^.

To our knowledge, all published studies attempting to understand the coordinated regulation of RPGs focus on the RPG promoter regions^6^,^9^,^12^,^15^,^18^,^19^,^20^,^21^,^22^,^23^,^24^,^25^. In this study, the promoter region of a gene is defined as the region from the upstream 1000 bp of its gene start site to the 100 bp downstream of its gene start site. Focusing on the gene promoter regions to understand gene regulation is limited, as the regulatory regions of mammalian genes can be located hundreds of thousands of bp away from the transcriptional start site of genes^26^. It is thus vital to study distal as well as proximal regulatory regions of human RPGs to have a better understanding of their coordinate expression pattern. Here and in the following, the proximal regions of a gene are regions that overlap with the promoter region of this gene, and the distal regions of a gene are regions that are at least 2.5 kilobase away from any gene and are within one megabase to this gene under consideration.

The high throughput data generated so far provide an unprecedented opportunity to study the transcriptional regulation of human RPGs. The DNase I hypersensitive sites (DHSs) delineate open chromatin regions under an experimental condition, which may indicate the locations of active regulatory regions under this condition^27^,^28^. The Hi-C and the chromatin interaction analysis by paired-end tag sequencing (ChIA-PET) describe the interacting genomic regions under an experimental condition, which can identify a large number of distal regulatory regions and their target genes under this condition^29^,^30^. Because RPGs are active under every normal condition, the generated DHS, Hi-C, ChIA-PET data, together with other types of data, provide the information of their distal and proximal regulatory regions under various conditions.

We integrated the aforementioned high throughput data to study the transcriptional regulation of the 80 human RPGs. We predicted 217 RPG regulatory regions, including 147 distal regions that are likely to modulate the expression of human RPGs. More than 86.6% of these regulatory regions overlapped with the regions that interact with RPG promoters identified by Hi-C or ChIA-PET. Computational motif analyses identified 31 non-redundant motifs in these predicted RPG regulatory regions, which included 57.1% of motifs that were known to regulate RPGs in literature. Interestingly, more than 93.5% of the identified motifs occurred in both distal and proximal regulatory regions predicted. In addition, we also observed that RPGs are likely to be differentially regulated in different cell types and tissues. Our study advances the understanding of how RPGs are co-ordinately modulated, which sheds light to the general principles of gene transcriptional regulation in mammals.

## Results

### RPGs are regulated by both distal and proximal regulatory regions

To predict regulatory regions, we investigated DHSs within one megabase of 80 human RPGs across 349 experiments^31^. DHSs were used because DHSs indicate open chromatin regions, where regulatory proteins in general bind to regulate their target genes. We assumed that a region was a possible RPG regulatory region, if it was within one megabase neighbourhood of a RPG and overlapped with DHSs across at least a large number of these 349 experiments. In other words, we assumed that every RPG was regulated in the same way across the majority of the 349 experiments. We considered one megabase neighbourhood of RPGs because the distance between enhancers and almost all of their experimentally validated target genes was within one megabase^26^. Moreover, the most adjacent protein-coding genes are within one megabase of all RPGs except RPS29.

**Figure 1:**
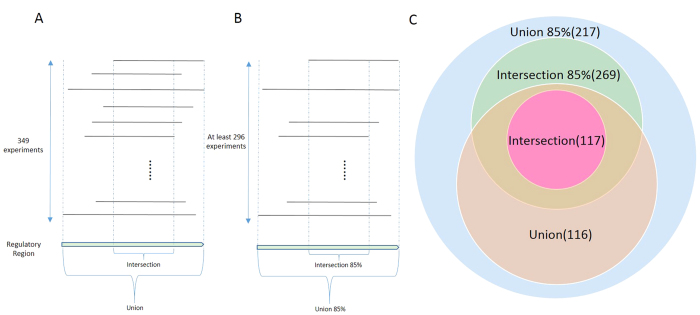
Four types of RPG regulatory regions. (**A**) The definition of intersection and union regions. Each row represents one of the 349 experiments. A solid line in a row (except the last row) represents a DHS region identified by Maurano *et al.*^31^ under the corresponding experimental conditions. The solid lines in the last row are the predicted RPG regulatory regions. (**B**) The definition of intersection 85% and union 85% regions. (**C**) The overlapping of the four types of RPG regulatory regions. There are 117 intersection regions, 116 union regions, 269 intersection 85% regions, and 217 union 85% regions identified.

Based on the number of experiments in which a DHS region overlapped with other DHS regions and the degree these DHS regions overlapped, we predicted four types of RPG regulatory regions: intersection, intersection 85%, union, and union 85% (Fig. 1, Supplementary Table S1, Figures S1–S4). An intersection region was a common regulatory region shared by all 349 experiments. In other words, every position in an intersection region was contained in at least one experimentally determined DHS region in each of the 349 experiments^31^. An intersection 85% region was a common regulatory region shared by at least 85% of the 349 experiments (i.e. 296 experiments). A union region was a region overlapping with at least one DHS region in each of the 349 experiments while any position in this union region may not be shared by DHSs in any two experiments. Similarly, a union 85% region was a region overlapping with at least one DHS region in each of a given *n* experiments while any position in this region may not be shared by DHSs in any two experiments, where *n* was no smaller than 296.

We predicted 117, 269, 116 and 217 intersection, intersection 85%, union, and union 85% RPG regulatory regions, respectively (Table 1). Each type of regions are associated with all RPGs except RPS17 and RPS4Y. Since RPS4Y was only present in samples from males and only a fraction of the 349 experiments were based on male samples, we excluded RPS4Y in the following analyses. Note that two regions predicted for RPS21 by intersection were shared by 348 experiments, one of which was only 24 bp away from a DHS in the remaining dataset. We believed that DHSs may not be defined perfectly, and considered the two regions as RPS21 intersection regions. This was one of the reasons that we computationally defined the other three types of regulatory regions.

We further compared the four types of predicted RPG regulatory regions (Fig. 1). Each type of region regulated 79 RPGs except RPS17. All 117 intersection regions were included in the 116 union regions, and all 269 intersection 85% regions were contained in the 217 union 85% regions, suggesting that the criteria to define union regions was looser than that of intersection regions. Intersection regions had the shortest average length while union regions had the longest average length. The much longer regions from the two types of union strategies suggested that many predicted regulatory regions were not shared across experiments and RPGs may be regulated differently under different conditions. In total, we identified 269 regulatory regions based on intersection and intersection 85%, and 217 regulatory regions based on union and union 85%.

Because RPGs are active under every experimental condition, we checked whether each type of predicted RPG regulatory regions overlapped with the promoter of every RPG. Surprisingly, we found that promoters of eight, five, four and four of the 79 RPGs did not overlap with any predicted intersection, intersection 85%, union, and union 85% regulatory regions, respectively (Table 1). The four RPGs without any predicted regulatory region overlapping with their promoters were RPL13, RPL40, RPL4, and RPS17, which had DHSs overlapped with their promoters in 104, 10, 1, and 1 experiment(s), respectively. Since RPL13 and RRL40 had proximal DHSs in 104 and 10 experiments, respectively, and did not have any predicted regulatory regions overlapping with their promoters, it is likely that at least certain RPGs might be regulated differently under different conditions. For RPL4 and RPS17, there was no DHS overlapping with their promoters in 348 experiments, suggesting that maybe these two genes were regulated differently without open promoter regions, or these 348 experiments failed to detect DHS signals in their promoters.

We also checked whether each type of predicted RPG regulatory region overlapped with the distal regions of every RPG. Recall a distal region of a RPG was a region that was within one megabase to this RPG and at least 2.5 kilobase away from this RPG and any other gene. From Table 1, it was evident that RPGs may be regulated by both distal and proximal regulatory regions. There was at least one predicted distal regulatory region for 63 RPGs. There existed both distal and proximal regulatory regions predicted for 60 RPGs. Under looser criteria (85%), the number of predicted distal regions was increasing while the number of predicted proximal regions was almost intact, suggesting that distal regions were likely condition-specific and only active in a fraction of these 349 experiments. This observation held when we continued to change our criteria from 85% to 1%: the number of proximal intersection regions was slightly increased from 78 to 93, while the number of distal intersection regions was greatly increased from 144 to 2787. Because the intersection 85% regions and the union 85% regions overlapped with more PRG promoters and had more reliable distal regulatory regions, we considered them as better collections of regulatory regions than those from intersection and union, respectively. In total, we predicted 269 and 271 regulatory regions based on intersection 85% and based on union 85%, repsectively. Because the 269 regions from intersection 85% were contained in the 217 regions from union 85%, we claimed that we identified 217 RPG regulatory regions.

### More than 86.6% of the predicted regulatory regions directly or indirectly interacted with RPG promoters in Hi-C or ChIA-PET experiments

To assess the quality of the predicted regulatory regions, we compared them with the candidate RPG regulatory regions from Hi-C and ChIA-PET experiments. We defined the candidate RPG regulatory regions as those that directly or indirectly interacted with RPG promoters in Hi-C experiments or ChIA-PET experiments with an antibody against RNA polymerase II. The regions indirectly interacting with RPGs, which we termed indirect regions, were those that interacted with regions directly interacting with RPG promoters. Because these candidate regulatory regions directly or indirectly interacted with RPG promoters, we hypothesized that these regions may be important RPG regulatory regions. Note that although these candidate regulatory regions likely regulated RPGs, their regulatory function was hypothetical and still needed experimental validation. We found that more than 86.6% of the predicted 217 regulatory regions overlapped with the candidate RPG regulatory regions.

We obtained the candidate RPG regulatory regions from seven cell types. From five Hi-C datasets in HUVEC, GM12878, NHEK, K562, and HMEC, and two ChIA-PET datasets in K562 and MCF7, we obtained 3053 and 955 candidate RPG regulatory regions, respectively. These candidate RPG regulatory regions included 3053 and 517 direct regions from Hi-C and ChIA-PET, respectively, and 1266 indirect regions from ChIA-PET. Note that after combining the overlapping candidate regulatory regions from ChIA-PET, we only obtained 955 non-redundant candidate regulatory regions. We did not consider the indirect candidate regions from Hi-C because the candidate regulatory regions identified from Hi-C were much longer and thus might not be as reliable as the indirect candidate regions from ChIA-PET. All RPGs except RPS17 had candidate regulatory regions, which agreed with our computational analyses. On average, a direct candidate region was 10456 bp long and an indirect candidate region was 10073 bp long (Supplementary Table S2).

We compared the predicted regulatory regions with the obtained candidate regulatory regions. We found that the majority of the computationally predicted regions overlapped with the candidate regulatory regions (Table 2). For instance, 114 of the 117 intersection regions and 113 of the 116 union regions overlapped with at least one candidate regulatory region. For the 217 predicted regulatory regions, 188 (86.6%) regions overlapped with the candidate regulatory regions. Similarly, we found that the majority of the computationally predicted distal regions overlapped with the candidate regulatory regions (Table 2). In contrast, we found that only 96 (18.6%) of the 517 direct candidate regulatory regions and only 111 (8.8%) of the 1266 indirect candidate regulatory regions from ChIA-PET overlapped with at least one of the computationally defined regulatory regions, suggesting that a large number of the candidate regulatory regions were condition-specific and were active in only a small subset of the 349 experiments.

To assess the significance of the overlap of the candidate regulatory regions and the computationally predicted (distal) regulatory regions, we randomly selected the same numbers of (distal) regions with the same corresponding sizes within the 1 megabase of the corresponding RPGs. We then compared the overlap of these simulated regions with the candidate regulatory regions. We found that no more than 8.2% of randomly generated (distal) regions in RPG neighbourhood overlapped with the candidate regulatory regions. The p-values of observing that the same number of predicted RPG regulatory regions overlapped with the candidate RPG regulatory regions were small (Table 2), suggesting that the predicted regions are likely functional and bona fide RPG regulatory regions, although the regulatory function of these candidate RPG regulatory regions still needs to be experimentally validated.

Because the candidate regulatory regions from ChIA-PET might have higher quality due to their much shorter lengths, we further investigated the overlap of the candidate regulatory regions from ChIA-PET with the computationally predicted (distal) RPG regulatory regions. We found that 92 of the 117 intersection regions and 106 of the 116 union regions overlapped with at least one of the ChIA-PET candidate regulatory regions. For the 217 predicted RPG regulatory regions, 158 (72.5%) regions overlapped with the ChIA-PET candidate regulatory regions (p-value = 4.6E-114). In contrast, no more than 9.2% of the 217 randomly generated (distal) regions in RPG neighbourhood overlapped with the ChIA-PET candidate regulatory regions, indicating that the computationally defined regulatory regions make sense and are likely to regulate RPGs under different conditions, although the regulatory function of the ChIA-PET candidate regulatory regions is not experimentally validated.

### Motifs identified in the predicted regulatory regions contained the majority of motifs known to regulate RPGs in literature

We studied motifs in the predicted regulatory regions. We applied a recently developed tool, SIOMICS^32^,^33^, for motif discovery due to its good performance on large sequence datasets^34^. SIOMICS predicted 14 and 27 motifs in the 269 intersection-based regions and in the 217 union-based regions, respectively (Supplementary Table S3, Methods). In total, 31 non-redundant motifs were predicted from the two sets of regions. We observed that almost all predicted motifs were similar to the known motifs. We also noticed that eight (57.1%) motifs known to regulate RPGs were included in our predicted motifs. Interestingly, 93.5% of the identified motifs occurred in both distal and proximal regulatory regions, and 40 of the 79 RPGs had at least one proximal regulatory region and one distal regulatory region that may be regulated by the same motifs.

We applied SIOMICS^32^,^33^ to the 269 intersection-based regions and the 217 union-based regions, respectively. We identified 14 and 27 motifs in these two sets of regions, respectively (Supplementary Table S3). We compared the predicted motifs with 1082 known motifs in JASPAR 2016 core database by combining two tools, TOMTOM^35^ and STAMP^36^ (Methods). We found that 13 of the 14 predicted motifs from the 269 intersection-based regions and 26 of the 27 predicted motifs from the 217 union-based regions were similar to the known motifs in JASPAR. The well-known RPG related motifs such as YY1, SP1 and GABPA motifs^6^ were included in the fourteen predicted motifs from the intersection-based regions, supporting the good quality of the predicted RPG regulatory regions.

Because motifs in JASPAR were not specifically for RPGs, we further compared predicted motifs with motifs known to regulate RPGs in literature. We went over more than 18 papers and collected 14 non-redundant experimentally validated motifs related to RPG regulation (Supplementary Table S4). We noticed that four (28.6%) and eight (57.1%) of these known RPG related motifs were included in the lists of the 14 and 27 predicted motifs, respectively. For instance, Parry *et al.* identified the TCT motif in human RPG promoters that was conserved from Drosophila to humans^37^. We found a motif matching the TCT motif in the 14 predicted motifs (TOMTOM E-value = 0.26 and STAMP E-value = 6.17e-4), and three of the 27 predicted motifs were similar to the TCT motif. The CTTTCC motif was observed in regulating several RPGs^38^. We found five similar motifs in the two lists of predicted motifs.

With the predicted motifs supported by literature, we studied their occurrence in the predicted RPG regulatory regions. We observed that the majority of the predicted motifs occurred in both distal regulatory regions and proximal regulatory regions: 12 of the 14 predicted motifs from intersection-based regions (binomial test p-value 0) and 24 of the 27 predicted motifs from union-based regions (binomial test p-value 0) occurred in both proximal and distal regions. The p-value of sharing motifs by proximal and distal regions based on intersection was much smaller than the p-value based on union, because the union-based regions were much longer than the intersection-based regions. At least the p-value of sharing motifs by proximal and distal regions based on intersection regions suggested that RPGs employed the same motifs in both proximal and distal regulatory regions. Moreover, at least one motif occurred in both proximal and distal regions of 40 RPGs (binomial test p-value 0). The above analyses suggested that RPGs likely employed the same motifs in both proximal and distal regulatory regions of the same RPGs, which may facilitate the interaction between distal and proximal regions of the same RPGs.

### RPGs may be regulated differently in different cell types

The above analyses were based on the assumption that the regulatory mechanisms of a RPG are similar across various experimental conditions. The assumption makes sense for the identification of common regulatory mechanisms across experiments. However, we are still left with the question whether RPGs are regulated the same or differently across different experimental conditions.

To address this question, we investigated the candidate RPG regulatory regions from the Hi-C experiments in five cell types (Methods). We identified 3053 candidate RPG regulatory regions, each at least 5000 bp long and interacting with at least one RPG promoter. Compared with the DHS regions from the 349 experiments^31^, surprisingly, only 1515 of the 3053 candidate regions overlapped with at least one DHS region. In other words, the majority of the 3053 candidate RPG regulatory regions did not have enriched DHS signals in any of the 349 experiments. Although these 349 experiments could not guarantee to identify all DHS regions under the corresponding conditions, it was showed that the sensitivity to identify a true DHS region by these experiments was larger than 98%^39^. Given such a sensitivity, it was unlikely that the remaining 1538 (=3503–1515) candidate regulatory regions did not have enriched DHS signals in 349 experiments, if they regulated RPGs in the majority of these experiments. It was thus evident that a large number of the candidate regulatory regions only regulated RPGs in certain experiments if they did regulate RPGs, suggesting that RPGs may be regulated differently in these experiments.

**Figure 2:**
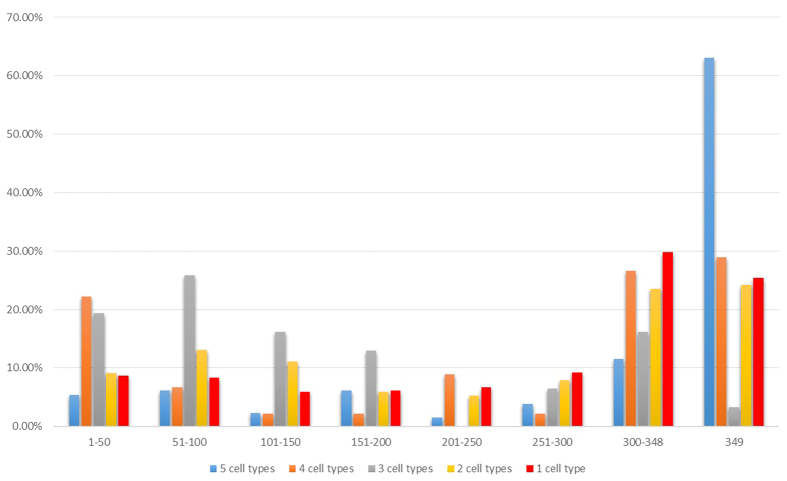
The DHS activity of the five classes of candidate RPG regulatory regions from Hi-C. Different colours represent different classes of candidate regulatory regions discovered in different numbers of cell types by Hi-C. The X axis represents the number of experiments where a candidate regulatory region had the DHS activity. The Y axis represents the percent of candidate regulatory regions among each type of candidate regulatory regions had the DHS activities in a given number of experiments by Maurano *et al.*^31^.

We further investigated the 1515 candidate regulatory regions that overlapped with at least one DHS region in the 349 experiments^31^. We divided these 1515 regions into five classes based on the number of cell types in which the Hi-C experiments identified the regions. The more cell types a region was independently identified by Hi-C, the more likely this region was functional. For every candidate regulatory region in each class, we identified the number of experiments in which it had DHS signals (Fig. 2). We found that although 359 (23.7%) of the 1515 candidate regions were identified in two cell types by Hi-C, 53 (14.8%) of the 359 candidate regions occurred in fewer than 20% of the 349 experiments. For instance, for the 130 candidate regions identified in all five cell types by Hi-C, 9 candidate regions had DHS signals in less than 20% of the 349 experiments, suggesting that at least a fraction of these highly-reliable candidate RPG regulatory regions were likely condition-specific and RPGs may be regulated differently under different conditions.

## Discussion

We studied the transcriptional regulation of human RPGs by integrating DHS, Hi-C, ChIA-PET, and other data. We predicted 217 regulatory regions that probably play important roles in modulating the expression of RPGs, and demonstrated that RPGs had both distal and proximal regulatory regions. More than 86.6% of the predicted regulatory regions overlapped with the candidate RPG regulatory regions from Hi-C or ChIA-PET experiments. Motif analyses on these predicted regions showed that distal regions and proximal regions of RPGs shared the same regulatory motifs. We also found that RPGs may be regulated differently across different experimental conditions, suggesting that the cell- or tissue-specific regulatory regions of RPGs still need to be identified. Our study shed new light on gene transcriptional regulation in mammals.

We demonstrated that the predicted distal and proximal regulatory regions of RPGs employ the same set of regulatory motifs. This conclusion is likely to hold because the majority of motifs known to regulate RPGs were included in our predicted motifs. Moreover, for three TFs, YY1, SP1, GABPA, that are known to regulate RPGs, we analysed their ChIP-seq (chromatin immunoprecipitation followed by massive parallel sequencing) data in GM12878, and found that ChIP-seq peaks of all three TFs overlapped with both distal and proximal RPG regulatory regions and we could find segments similar to the corresponding motifs in these regions (Supplementary Table S5). We also applied other methods such as DREME^40^ to identify motifs and found the identified motifs were in general similar to our predicted motifs, further supporting our conclusion. For instance, 86.7% of identified DREME motifs were similar to the predicted 27 motifs (Supplementary Table S4).

We showed that RPGs may be regulated differently under different experimental conditions. First, at least a portion of the identified intersection 85% regions and the identified union regions were not shared across the 349 experiments. Second, when we lowered our criteria to define intersection regions in 100% to 1% of the 349 experiments, the number of the identified proximal regulatory regions was almost intact after we considered 85% of the experiments while the number of the identified distal regulatory regions was always increasing, suggesting many regulatory regions may only regulate RPGs in a portion of these experiments. Third, at least a fraction of the candidate RPG regulatory regions from Hi-C also showed condition-specific DHS activities. Therefore, there may be other cell- or tissue-specific RPG regulatory regions to be identified.

We summarized the location distribution of the intronic RPG regulatory regions we predicted in the Supplementary Table S6. More than 81.3% of these intronic RPG regulatory regions overlapped with the RPG promoter regions and were considered as proximal regulatory regions (Table 1). The majority of the remaining intronic regulatory regions were quite far from the gene start sites, with the average distance to the corresponding gene start sites larger than 10.2 kilobases (Supplementary Tables S6 and S7).

We did not predict any regulatory region for RPS17 from computational or experimental data analyses. In fact, in only one of the 349 experiments, was there an identified DHS region overlapping with the promoter of RPS17. We suspect that RPS17 might be annotated incorrectly at the National Center for Biotechnology Information (NCBI). The NCBI server indicates that “RPS17 is also known as RPS17L”. However, the locations of RPS17L and RPS17 at NCBI are different. Interestingly, the Ensembl genome browser annotated RPS17 as RPS17L using the GENCODE information (http://www.gencodegenes.org/) while the GENCODE annotated RPS17 and RPS17L similarly as NCBI. We also tried to use RPS17L to replace RPS17 and we still could not predict a regulatory region for RPS17L. It is also possible that RPS17 is annotated correctly at NCBI, while reads from the RPS17 promoter are not readily mappable. In fact, the average mappability value of its promoter was 0.46 (http://hgdownload.cse.ucsc.edu/gbdb/hg19/bbi/wgEncodeCrgMapabilityAlign24mer.bw), lower than the average mappability of promoters of the 80 RPGs, 0.7.

We did not predict any regulatory region overlapping with the promoters of the following four RPGs, RPL13, RPL40, RPL4, and RPS17. The average mappability values of the promoters of the four genes were 0.5, 0.35, 0.81, and 0.46, respectively. Because the mappability of the RPL4 promoter was larger than the average mappability of the 80 RPG promoters, mappability alone may not be able to explain why RPL4 and other three RPGs did not have predicted proximal regulatory regions. In fact, the RPS10 promoter had an average mappability no larger than that of any of these four RPG promoters, while RPS10 had an identified proximal regulatory region (Supplementary Table S8). It is thus likely that the four RPGs may be regulated differently from other RPGs without open promoter regions, or the 349 experiments failed to identify the DHS activity in their promoter regions.

Although this is the first time that a large number of distal RPG regulatory regions have been identified, there are still many unknowns. We narrowed our analyses within 1 megabase genomic regions to RPGs, because 96.5% of the candidate regulatory regions from Hi-C were within 1 megabase neighbourhood of RPGs or their target genes^39^,^41^,^42^,^43^,^44^,^45^. However, there might be more distant RPG regulatory regions. In addition, we only considered the regulatory regions shared by a large number of experiments and demonstrated that there may exist other condition- and tissue-specific RPG regulatory regions. Finally, although we predicted the shared regulatory regions across experiments, how these regulatory regions together with cell- and tissue-specific regulatory regions modulate RPG expression was not explored. We are working on these and other directions to improve our understanding of human RPG transcriptional regulation.

## Methods

### Experimental data used

We downloaded 349 DHS datasets from Maurano *et al.*^31^. These 349 datasets were generated by DNase I hypersensitive analysis followed by sequencing (DNase-seq) experiments in 31 major tissues and cell types. DNase-seq experiments define open chromatin regions called DHS regions under an experimental condition, which may potentially contain active regulatory regions under this condition^27^,^28^. There were 70,474 to 337,456 DHS regions defined in each dataset by Maurano *et al.* with the false discovery rate (FDR) of 0.05^31^.

We downloaded two ChIA-PET datasets in MCF7 and K562 from GSE33664 that contained long range interactions associating with RNA polymerase II^46^. The ChIA-PET interacting regions were generated with the following criteria: (a) PET count >2 for each PET cluster from the pilot libraries, and PET count >3 for each PET cluster from the saturated libraries; (b) FDR < 0.05; and (c) the genomic span of interacting regions is from 8 kilobases to 1 megabases. Three additional ChIA-PET datasets were generated by the same lab. We used the two instead of the additional three datasets because of the much higher sequencing depth in the two datasets we used. From the two ChIA-PET datasets, we collected 517 direct regions that interacted with RPG promoters. We called these regions as direct candidate regulatory regions of RPGs. We also collected 1266 indirect candidate regions that interacted with the direct regulatory regions of RPGs, which we termed as indirect candidate RPG regulatory regions. In total, we identified 955 candidate regulatory regions from ChIA-PET experiments after merging overlapping regions (Supplementary Table S2).

We downloaded five Hi-C datasets with the resolution of five kilobases in K562, HMEC, HUVEC, GM12878 and NHEK from GSE63525^47^. There were other Hi-C data available. We only used the Hi-C data from the above five cell lines because of their higher resolution, more available cell types, and because they were generated with the same pipelines. The downloaded files are raw contact matrices. Using the normalization equation from Rao *et al.*^47^, the raw contact matrices were normalized. To find a significant interactivity between two regions, a cut-off was needed to select pairs of interacting regions from the normalized contact matrices. Our rule of the cut-off selection was to select the largest cut-off so that as many as possible RPGs have at least one region interacting with their promoters under this cut-off. The rationale behind this rule is that RPGs are active under every experimental condition and their potential distal regulatory regions should interact with their promoters under each condition. Moreover, the ChIA-PET data clearly showed that all RPGs except RPS17 and RPS4Y had at least one direct candidate distal regulatory region. We selected a cut-off of 30 for all five cell lines, which was much larger than the average normalized contacts in the five cell lines. With this cut-off, we obtained 421, 190, 157, 3048 and 235 regions that interacted with RPG promoters in K562, HMEC, HUVEC, GM12878 and NHEK, respectively (Supplementary Table S2).

We downloaded the ChIP-seq datasets for SP1, GABPA, and YY1 in GM12878 from http://hgdownload.cse.ucsc.edu/goldenPath/hg19/encodeDCC/wgEncodeAwgTfbsUniform/. We checked whether the predicted TF binding sites were within the ChIP-seq peaks defined in these datasets and whether the binding sites were in the distal or proximal regulatory regions of RPGs.

### Motif analyses in regulatory regions

With the predicted regulatory regions, we extended them so that each region was at least 800 bp long. The reason to extend these regions was that DHS regions may be defined imperfectly and a typical regulatory region is in general contained in a region around 800 bp long^34^,^48^,^49^. We extracted the corresponding DNA sequences in these extended regions and masked the repeats in these sequences. The resulted sequences were input into SIOMICS^32^,^33^ for motif discovery, with the following parameters “-w 8 -m 100 -s 5 -c 0.01 -r 20”. We applied SIOMICS to the sequences from the 269 regulatory regions identified by intersection 85% and from the 217 regulatory regions identified by union 85%, respectively. The motifs predicted from the two sequence datasets were compared and combined into a set of non-redundant motifs, which were considered as the predicted motifs. We also applied DREME^40^ to the same sequence datasets with the following parameters “-e 0.05 –mink 8 –maxk 12”.

### Motif similarity comparison

We compared motifs with the following two tools, TOMTOM^35^ and STAMP^36^. The two tools were widely used for motif similarity comparison. Our previous study showed that neither of the two tools was perfect, and combining them achieved an improved accuracy^34^. The same as in our previous study, we claimed that two motifs were similar if their TOMTOM E-value was smaller than 0.5 and their STAMP E-Value was smaller than 1E-4, or their TOMTOM E-value was smaller than 1 and their STAMP E-value was smaller than 1E-5.

## Additional Information

**How to cite this article**: Li, X. *et al.* Integrative analyses shed new light on human ribosomal protein gene regulation. *Sci. Rep.* **6**, 28619; doi: 10.1038/srep28619 (2016).

## Supplementary Material

Supplementary Information

Supplementary table S1

Supplementary table S2

Supplementary table S3

supplementary table s4

supplementary table s5

supplementary table s8

## Figures and Tables

**Table 1:** Four types of RPG regulatory regions.

	Intersection	Intersection 85%	Union	Union 85%
#Regulatory regions predicted	117	269	116	217
Average length	298bp	444bp	4769bp	3785bp
Average distance to gene	12989bp	28051bp	14901bp	32919bp
#Regions overlapping with RPG proximal regions	73	78	76	77
#RPGs with identified proximal regions	71	74	75	75
#Distal regions identified	31	144	52	147
#Regions in RPGs introns	66	97	82	91
Max# regulatory regions identified for a RPG	4(RPL11)	13(RPL11)	4(RPL11)	10(RPL11)
#RPGs with only one identified regulatory region	43 genes	11 genes	46 genes	18 genes
#Genes with both distal and proximal regions	25	54	36	60

One common regulatory region predicted for RPS11 and RPL13A was considered separately for these two genes from the third to the last row. RPS17, RPS27A, RPL4, RPL13, RPL40, RPLP2, RPL6 and RPL27A did not have any intersection regulatory region overlapping with their promoter. RPS17, RPL4, RPL13, RPL40, and RPLP2 did not have any intersection 85% region overlapping with their promoters. RPS17, RPL4, RPL13, RPL40 did not have any predicted regulatory region overlapping with their promoters.

**Table 2:** Identified regulatory regions were supported by experiments.

	Intersection	Intersection 85%	Union	Union 85%
#Predicted regions	118 (31)	270 (144)	117 (52)	218 (147)
#Predicted regions overlapping with Hi-C candidate regions	114 (27)	248 (122)	114 (49)	187 (116)
#Predicted regions overlapping with ChIA-PET candidate regions	93 (25)	196 (93)	107 (46)	158 (91)
#Predicted regions overlapping with candidate regions	115 (28)	250 (124)	114 (49)	189 (118)
#Random regions overlapping with Hi-C candidate regions	7 (2)	21 (12)	9 (8)	16 (12)
#Random regions overlapping with ChIA-PET candidate regions	6 (2)	31 (10)	14 (8)	20 (11)
#Random regions overlapping with candidate regions	11 (3)	37 (18)	17 (12)	26 (14)
Significance of the predicted regions overlapping with Hi-C candidate regions	1.85E-136 (1.74E-30)	1.23E-246 (2.66E-109)	4.56E-125 (2.16E-38)	3.79E-178 (6.36E-98)
Significance of the predicted regions overlapping with ChIA-PET candidate regions	4.93E-098 (1.39E-26)	1.40E-122 (6.21E-72)	6.94E-088 (7.13E-33)	4.62E-114 (4.14E-65)
Significance of the predicted regions overlapping with candidate regions	1.65E-116 (1.48E-27)	9.22E-190 (2.55E-91)	2.27E-093 (1.14E-29)	1.62E-142 (1.9E-93)

Significance was calculated by binomial tests. One common regulatory region predicted for RPS11 and RPL13A was considered separately for these two genes. The numbers in the parentheses were for the distal regulatory or random regions.

## References

[b1] Rodnina M. & Wintermeyer W. The ribosome as a molecular machine: the mechanism of tRNAmRNA movement in translocation. Biochemical Society Transactions 39, 658 (2011). 21428957 10.1042/BST0390658

[b2] Uechi T., Tanaka T. & Kenmochi N. A complete map of the human ribosomal protein genes: assignment of 80 genes to the cytogenetic map and implications for human disorders. Genomics 72, 223–230 (2001). 11401437 10.1006/geno.2000.6470

[b3] Kozak M. The scanning model for translation: an update. The Journal of cell biology 108, 229–241 (1989). 2645293 10.1083/jcb.108.2.229 PMC2115416

[b4] Donahue T. F. 12 Genetic Approaches to Translation Initiation in Saccharomyces cerevisiae. Cold Spring Harbor Monograph Archive 39, 487–502 (2000).

[b5] Hershey J. W. & Merrick W. C. 2 The Pathway and Mechanism of Initiation of Protein Synthesis. Cold Spring Harbor Monograph Archive 39, 33–88 (2000).

[b6] Perry R. P. The architecture of mammalian ribosomal protein promoters. BMC evolutionary biology 5, 15, 10.1186/1471-2148-5-15 (2005). 15707503 PMC554972

[b7] Hariharan N., Kelley D. E. & Perry R. P. Equipotent mouse ribosomal protein promoters have a similar architecture that includes internal sequence elements. Genes & development 3, 1789–1800 (1989). 2606348 10.1101/gad.3.11.1789

[b8] Meyuhas O. & Perry R. P. Construction and identification of cDNA clones for mouse ribosomal proteins: application for the study of r-protein gene expression. Gene 10, 113–129 (1980). 6993285 10.1016/0378-1119(80)90129-8

[b9] Kenmochi N. *et al.* A map of 75 human ribosomal protein genes. Genome research 8, 509–523 (1998). 9582194 10.1101/gr.8.5.509

[b10] Lascaris R. F., Mager W. H. & Planta R. J. DNA-binding requirements of the yeast protein Rap1p as selected *in silico* from ribosomal protein gene promoter sequences. Bioinformatics 15, 267–277 (1999). 10320394 10.1093/bioinformatics/15.4.267

[b11] Lieb J. D., Liu X., Botstein D. & Brown P. O. Promoter-specific binding of Rap1 revealed by genome-wide maps of protein–DNA association. Nature genetics 28, 327–334 (2001). 11455386 10.1038/ng569

[b12] Hu H. & Li X. Transcriptional regulation in eukaryotic ribosomal protein genes. Genomics 90, 421–423, 10.1016/j.ygeno.2007.07.003 (2007). 17707610

[b13] Wagner M. & Perry R. P. Characterization of the multigene family encoding the mouse S16 ribosomal protein: strategy for distinguishing an expressed gene from its processed pseudogene counterparts by an analysis of total genomic DNA. Molecular and cellular biology 5, 3560–3576 (1985). 3915781 10.1128/mcb.5.12.3560-3576.1985 PMC369187

[b14] Hariharan N., Kelley D. E. & Perry R. P. Delta, a transcription factor that binds to downstream elements in several polymerase II promoters, is a functionally versatile zinc finger protein. Proceedings of the National Academy of Sciences 88, 9799–9803 (1991). 10.1073/pnas.88.21.9799 PMC52808 1946404

[b15] Li X., Zhong S. & Wong W. H. Reliable prediction of transcription factor binding sites by phylogenetic verification. Proceedings of the National Academy of Sciences of the United States of America 102, 16945–16950, 10.1073/pnas.0504201102 (2005). 16286651 PMC1283155

[b16] Marion R. M. *et al.* Sfp1 is a stress-and nutrient-sensitive regulator of ribosomal protein gene expression. Proceedings of the National Academy of Sciences of the United States of America 101, 14315–14322 (2004). 15353587 10.1073/pnas.0405353101 PMC521938

[b17] Boon K. *et al.* N‐myc enhances the expression of a large set of genes functioning in ribosome biogenesis and protein synthesis. The EMBO journal 20, 1383–1393 (2001). 11250904 10.1093/emboj/20.6.1383 PMC145518

[b18] Li X. & Wong W. H. Sampling motifs on phylogenetic trees. Proceedings of the National Academy of Sciences of the United States of America 102, 9481–9486 (2005). 15983378 10.1073/pnas.0501620102 PMC1160516

[b19] Yoshihama M. *et al.* The human ribosomal protein genes: sequencing and comparative analysis of 73 genes. Genome research 12, 379–390, 10.1101/gr.214202 (2002). 11875025 PMC155282

[b20] Rhoads D., Dixit A. & Roufa D. Primary structure of human ribosomal protein S14 and the gene that encodes it. Molecular and cellular biology 6, 2774–2783 (1986). 3785212 10.1128/mcb.6.8.2774-2783.1986 PMC367844

[b21] Kraakman L. S., Mager W. H., Maurer K. T., Nieuwint R. T. & Planta R. J. The divergently transcribed genes encoding yeast ribosomal proteins L46 and S24 are activated by shared RPG-boxes. Nucleic acids research 17, 9693–9706 (1989). 2602141 10.1093/nar/17.23.9693 PMC335207

[b22] Hariharan N. & Perry R. P. A characterization of the elements comprising the promoter of the mouse ribosomal protein gene RPS16. Nucleic acids research 17, 5323–5338 (1989). 2762128 10.1093/nar/17.13.5323 PMC318113

[b23] Kenmochi N., Maeda N. & Tanaka T. The structure and complete sequence of the gene encoding chicken ribosomal protein L5. Gene 119, 215–219 (1992). 1398102 10.1016/0378-1119(92)90274-s

[b24] Toku S. & Tanaka T. A characterization of transcriptional regulatory elements in chicken ribosomal protein L37a gene. European journal of biochemistry 238, 136–142 (1996). 8665930 10.1111/j.1432-1033.1996.0136q.x

[b25] Ma X., Zhang K. & Li X. Evolution of Drosophila ribosomal protein gene core promoters. Gene 432, 54–59 (2009). 19059316 10.1016/j.gene.2008.10.025 PMC3232064

[b26] Lettice L. A. *et al.* A long-range Shh enhancer regulates expression in the developing limb and fin and is associated with preaxial polydactyly. Human molecular genetics 12, 1725–1735 (2003). 12837695 10.1093/hmg/ddg180

[b27] Song L. & Crawford G. E. DNase-seq: a high-resolution technique for mapping active gene regulatory elements across the genome from mammalian cells. Cold Spring Harbor Protocols 2010, pdb. prot5384 (2010). 10.1101/pdb.prot5384 PMC3627383 20150147

[b28] Crawford G. E. *et al.* Genome-wide mapping of DNase hypersensitive sites using massively parallel signature sequencing (MPSS). Genome research 16, 123–131 (2006). 16344561 10.1101/gr.4074106 PMC1356136

[b29] Lieberman-Aiden E. *et al.* Comprehensive mapping of long-range interactions reveals folding principles of the human genome. Science 326, 289–293, 10.1126/science.1181369 (2009). 19815776 PMC2858594

[b30] Fullwood M. J. *et al.* An oestrogen-receptor-alpha-bound human chromatin interactome. Nature 462, 58–64, 10.1038/nature08497 (2009). 19890323 PMC2774924

[b31] Maurano M. T. *et al.* Systematic localization of common disease-associated variation in regulatory DNA. Science 337, 1190–1195 (2012). 22955828 10.1126/science.1222794 PMC3771521

[b32] Ding J., Hu H. & Li X. SIOMICS: a novel approach for systematic identification of motifs in ChIP-seq data. Nucleic acids research 42, e35–e35 (2014). 24322294 10.1093/nar/gkt1288 PMC3950686

[b33] Ding J., Dhillon V., Li X. & Hu H. Systematic discovery of cofactor motifs from ChIP-seq data by SIOMICS. Methods 79, 47–51 (2015). 25171961 10.1016/j.ymeth.2014.08.006

[b34] Zheng Y., Li X. & Hu H. Comprehensive discovery of DNA motifs in 349 human cells and tissues reveals new features of motifs. Nucleic acids research gku1261 (2014). 10.1093/nar/gku1261 PMC4288161 25505144

[b35] Gupta S., Stamatoyannopoulos J. A., Bailey T. L. & Noble W. S. Quantifying similarity between motifs. Genome biology 8, R24 (2007). 17324271 10.1186/gb-2007-8-2-r24 PMC1852410

[b36] Mahony S. & Benos P. V. STAMP: a web tool for exploring DNA-binding motif similarities. Nucleic acids research 35, W253–W258 (2007). 17478497 10.1093/nar/gkm272 PMC1933206

[b37] Parry T. J. *et al.* The TCT motif, a key component of an RNA polymerase II transcription system for the translational machinery. Genes & development 24, 2013–2018 (2010). 20801935 10.1101/gad.1951110 PMC2939363

[b38] Wool I. G., Chan Y.-L. & Glück A. Structure and evolution of mammalian ribosomal proteins. Biochemistry and Cell Biology 73, 933–947 (1995). 8722009 10.1139/o95-101

[b39] Thurman R. E. *et al.* The accessible chromatin landscape of the human genome. Nature 489, 75–82 (2012). 22955617 10.1038/nature11232 PMC3721348

[b40] Bailey T. L. DREME: motif discovery in transcription factor ChIP-seq data. Bioinformatics 27, 1653–1659 (2011). 21543442 10.1093/bioinformatics/btr261 PMC3106199

[b41] Chepelev I., Wei G., Wangsa D., Tang Q. & Zhao K. Characterization of genome-wide enhancer-promoter interactions reveals co-expression of interacting genes and modes of higher order chromatin organization. Cell research 22, 490–503 (2012). 22270183 10.1038/cr.2012.15 PMC3292289

[b42] Heintzman N. D. *et al.* Histone modifications at human enhancers reflect global cell-type-specific gene expression. Nature 459, 108–112 (2009). 19295514 10.1038/nature07829 PMC2910248

[b43] Sanyal A., Lajoie B. R., Jain G. & Dekker J. The long-range interaction landscape of gene promoters. Nature 489, 109–113 (2012). 22955621 10.1038/nature11279 PMC3555147

[b44] He B., Chen C., Teng L. & Tan K. Global view of enhancer–promoter interactome in human cells. Proceedings of the National Academy of Sciences 111, E2191–E2199 (2014). 10.1073/pnas.1320308111 PMC4040567 24821768

[b45] Ernst J. *et al.* Mapping and analysis of chromatin state dynamics in nine human cell types. Nature 473, 43–49 (2011). 21441907 10.1038/nature09906 PMC3088773

[b46] Li G. *et al.* Extensive promoter-centered chromatin interactions provide a topological basis for transcription regulation. Cell 148, 84–98 (2012). 22265404 10.1016/j.cell.2011.12.014 PMC3339270

[b47] Rao S. S. *et al.* A 3D map of the human genome at kilobase resolution reveals principles of chromatin looping. Cell 159, 1665–1680 (2014). 25497547 10.1016/j.cell.2014.11.021 PMC5635824

[b48] Blanchette M. *et al.* Genome-wide computational prediction of transcriptional regulatory modules reveals new insights into human gene expression. Genome research 16, 656–668 (2006). 16606704 10.1101/gr.4866006 PMC1457048

[b49] Cai X. *et al.* Systematic identification of conserved motif modules in the human genome. BMC genomics 11, 567 (2010). 20946653 10.1186/1471-2164-11-567 PMC3091716

