# Supplementary material for: Integrative analyses shed new light on human ribosomal protein gene regulation
Source: Sci Rep. 2016 Jun 27;6:28619. doi: 10.1038/srep28619 (PMC4921865; doi:10.1038/srep28619)
Supplement: Supplementary Information [file srep28619-s1.pdf]

Supplementary Information: Integrative analyses shed new light on human ribosomal protein gene regulation

Xin Li[1, §], Yiyu Zheng[1, §], Haiyan Hu[1, *], Xiaoman Li[2, *]

[1]Department of Electrical Engineering & Computer Science, University of Central Florida, Orlando, FL, 32816, USA

[2]Burnett School of Biomedical Science, University of Central Florida, Orlando, FL, 32816, USA

[§]These authors contributed equally to this work

*Corresponding authors

Email addresses:

XinL: xli042@knights.ucf.edu

YZ: zhengyiyu@knights.ucf.edu

HH: haihu@cs.ucf.edu

XL: xiaoman@mail.ucf.edu

Supplementary tables and legends:

Supplementary Table S1: The four types of predicted RPG regulatory regions.

Supplementary Table S2: 1. Direct candidate RPG regulatory regions from Hi-C. Direct and indirect candidate RPG regulatory regions from ChIA-PET.

Supplementary Table S3: Motifs identified in intersection 85% and union 85% regions. 14 motifs identified in the intersection 85% regions and 27 motifs identified in the union 85% regions. All 41 motifs are in PWM format.

Supplementary Table S4: Predicted motifs compare with motifs from literature and from DREME predictions. Comparisons of the predicted motifs with motifs from literature and from the motifs predicted by DREME.

Supplementary Table S5: Predicted TFBSs compared with ChIP-seq experiments. Predicted TFBSs of the predicted motifs that were similar to YY1, SP1, GABAP motifs compared with the YY1, SP1, GABAP ChIP-seq data.

Supplementary Table S6: Location distribution of the predicted intronic RPG regulatory regions.

	Intersection	Intersection 85%	Union	Union 85%
#Identified regions overlapped with both intron and TSS	59	73	74	74
#Identified regions overlapped with intron only	7	24	8	17
#Total distal regions overlapped TSS or intron	66	97	82	91
Average distance to TSS(Region overlapped with intron only)	7231bp	5425bp	11313bp	10200bp
#RPGs whose regions overlapped with intron only	7	16	7	12

Supplementary Table S7: The predicted intronic RPG regulatory regions which are not overlapped with RPG TSS.

Gene Name	Start	End	Distance to TSS	Source
RPL6	112847213	112847505	9142	Intersection
RPL4	66797416	66797808	19062	Intersection
RPL10	153627183	153627314	612	Intersection

RPL13	89626874	89627158	13550	Intersection
RPL27A	8704135	8704383	140	Intersection
RPS27A	55459374	55459848	335	Intersection
RPL40(UBA52)	18682353	18682691	7777	Intersection
RPS11	50000641	50000737	1019	Intersection 85%
RPL6	112846863	112847560	9087	Intersection 85%
RPL6	112849467	112849556	7091	Intersection 85%
RPL3	39712396	39712698	2972	Intersection 85%
RPS6	19379729	19379729	543	Intersection 85%
RPS3	75119007	75119155	8472	Intersection 85%
RPLP2	809092	810073	251	Intersection 85%
RPLP2	819298	819839	10457	Intersection 85%
RPLP2	820024	820300	11183	Intersection 85%
RPL4	66796911	66798085	18785	Intersection 85%
RPS24	79807092	79807436	13574	Intersection 85%
RPL10	153627059	153627387	488	Intersection 85%
RPL13	89623889	89624122	10565	Intersection 85%
RPL13	89626621	89627284	13297	Intersection 85%
RPL39	118924963	118925186	436	Intersection 85%
RPL39	118925189	118925191	431	Intersection 85%
RPL37	40834623	40834727	660	Intersection 85%
RPL37	40834735	40834737	650	Intersection 85%
RPL35	127623725	127623731	509	Intersection 85%
RPL35	127623767	127623998	242	Intersection 85%
RPL35	127624001	127624012	228	Intersection 85%
RPL31	101629235	101629399	10544	Intersection 85%
RPL36	5691393	5691764	1121	Intersection 85%
RPL40(UBA52)	18682186	18682795	7610	Intersection 85%
RPL6	112846432	112848355	8292	Union
RPL26	8280028	8281146	5419	Union
RPLP2	818272	822815	9431	Union
RPL4	66791765	66798757	18113	Union
RPL4	66788746	66791749	25121	Union
RPL13	89625201	89633276	11877	Union
RPL38	72204945	72209695	5150	Union
RPL40(UBA52)	18681681	18684525	7105	Union
RPS10	34381813	34386851	7051	Union 85%
RPL6	112846432	112848355	8292	Union 85%
RPL6	112848731	112849709	6938	Union 85%
RPS19	42371874	42378522	7886	Union 85%
RPS3	75116346	75120220	5811	Union 85%

RPL26	8280028	8281146	5419	Union 85%
RPLP2	818272	822815	9431	Union 85%
RPLP2	823325	832951	14484	Union 85%
RPL4	66791765	66798757	18113	Union 85%
RPL4	66788746	66791749	25121	Union 85%
RPS24	79803034	79805514	9516	Union 85%
RPS24	79806416	79810156	12898	Union 85%
RPL13	89622873	89624744	9549	Union 85%
RPL13	89625201	89633276	11877	Union 85%
RPL38	72204945	72209695	5150	Union 85%
RPL31	101627451	101630128	8760	Union 85%
RPL40(UBA52)	18681681	18684525	7105	Union 85%

Supplementary Table S8: Mappability of RPG promoters. The mappability of the RPG promoters.

Supplementary Figures:

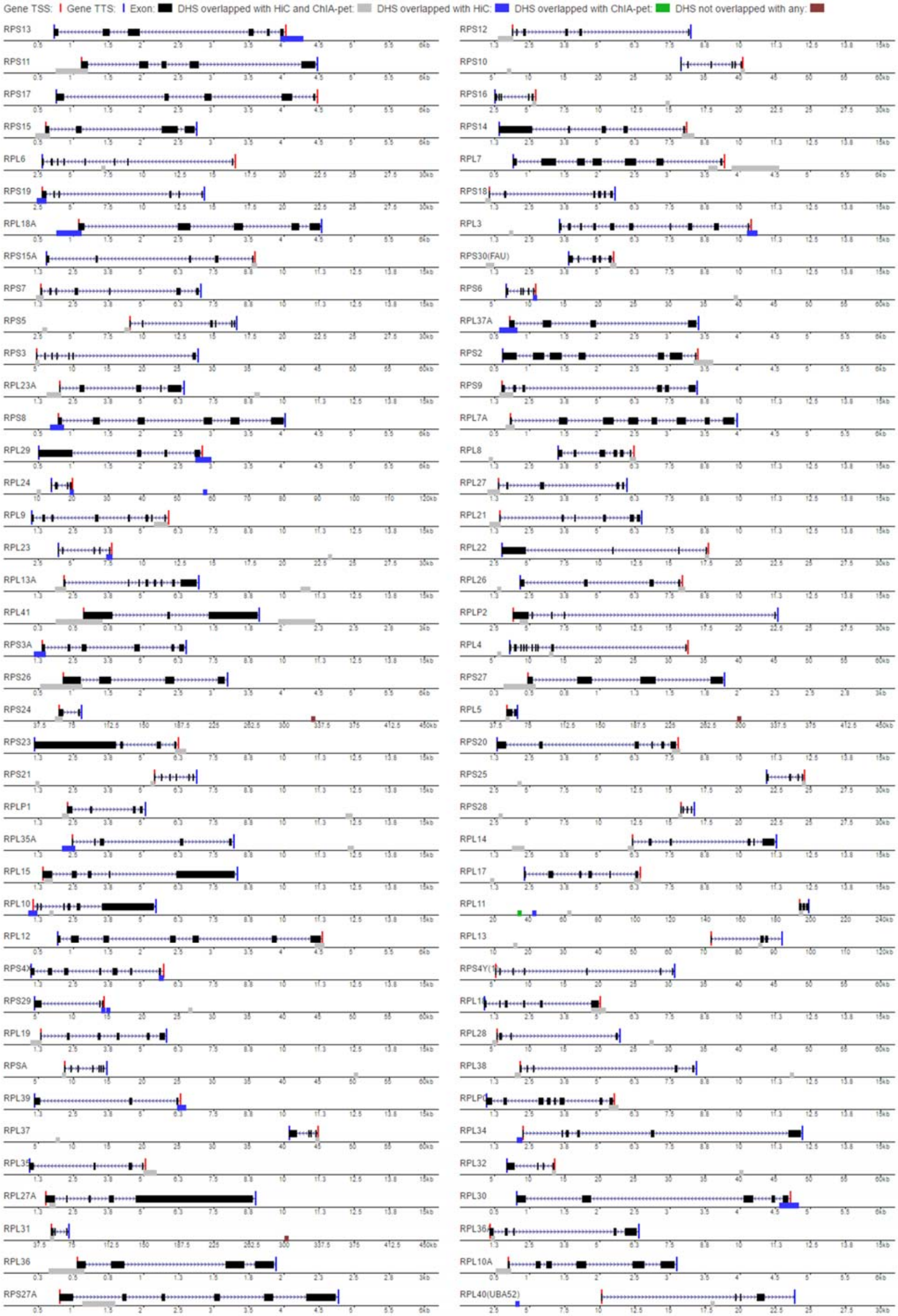

Figure S1. The predicted intersection regions. Each gene is shown in one row, followed by the ruler and the predicted regulatory regions.

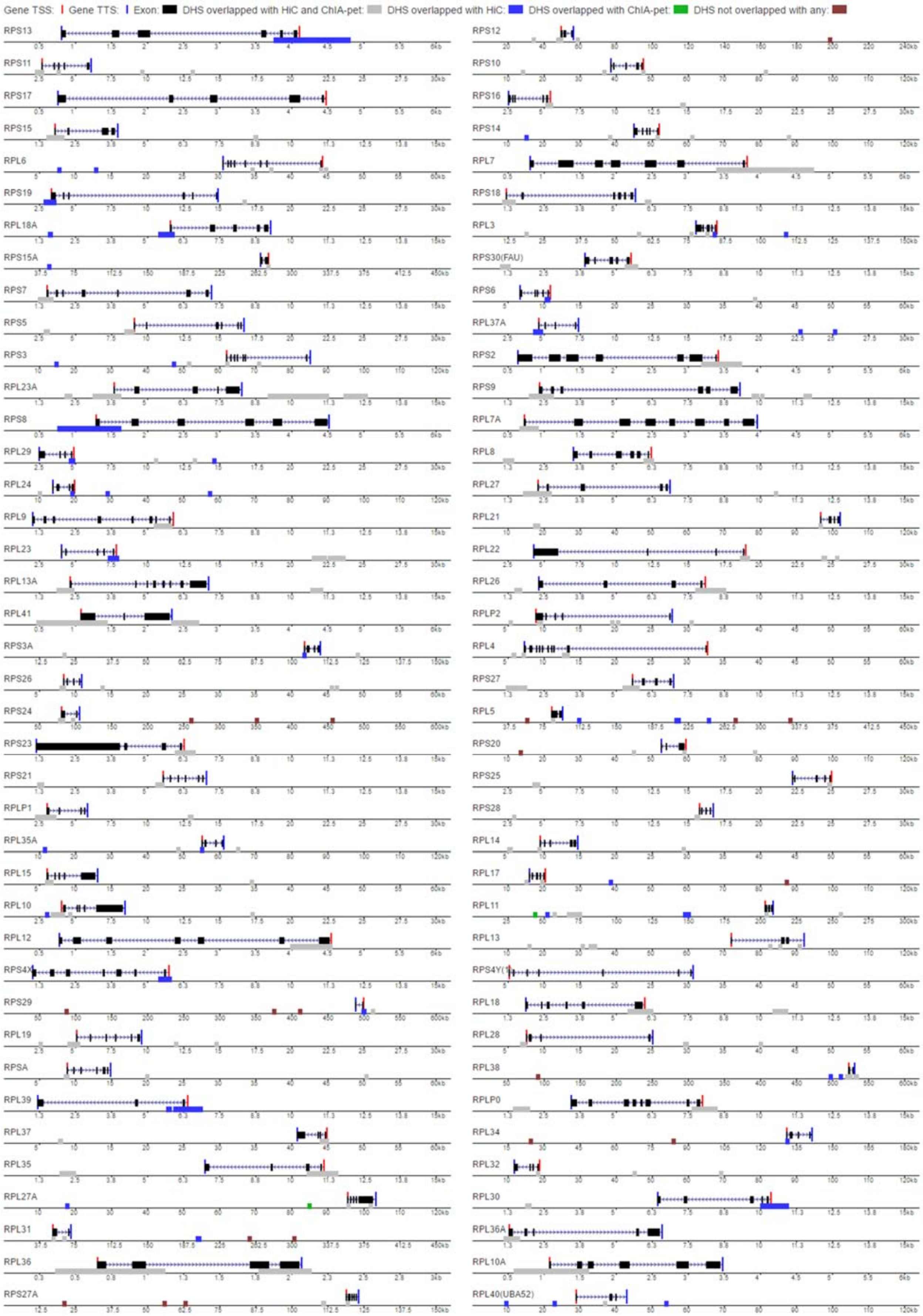
Gene TSS: Gene TTS: Exon: DHS overlapped with HiC and ChIA-pet: DHS overlapped with HiC: DHS overlapped with ChIA-pet: DHS not overlapped with any:
RPS13
RPS11
RPS17
RPS15
RPL6
RPS19
RPL18A
RPS15A
RPS7
RPS5
RPS3
RPL23A
RPS8
RPL29
RPL24
RPL9
RPL23
RPL13A
RPL41
RPS3A
RPS26
RPS24
RPS23
RPS21
RPLP1
RPL35A
RPL15
RPL10
RPL12
RPS4X
RPS29
RPL19
RPSA
RPL39
RPL37
RPL35
RPL27A
RPL31
RPL36
RPS27A
RPS12
RPS10
RPS16
RPS14
RPL7
RPS18
RPL3
RPS30(FAU)
RPS6
RPL37A
RPS2
RPS9
RPL7A
RPL8
RPL27
RPL21
RPL22
RPL26
RPLP2
RPL4
RPS27
RPL5
RPS20
RPS25
RPS28
RPL14
RPL17
RPL11
RPL13
RPS4Y(1
RPL18
RPL28
RPL38
RPLP0
RPL34
RPL32
RPL30
RPL36A
RPL10A
RPL40(UBA52)

Figure S2. The predicted intersection 85% regions. Each gene is shown in one row, followed by the ruler and the predicted regulatory regions.

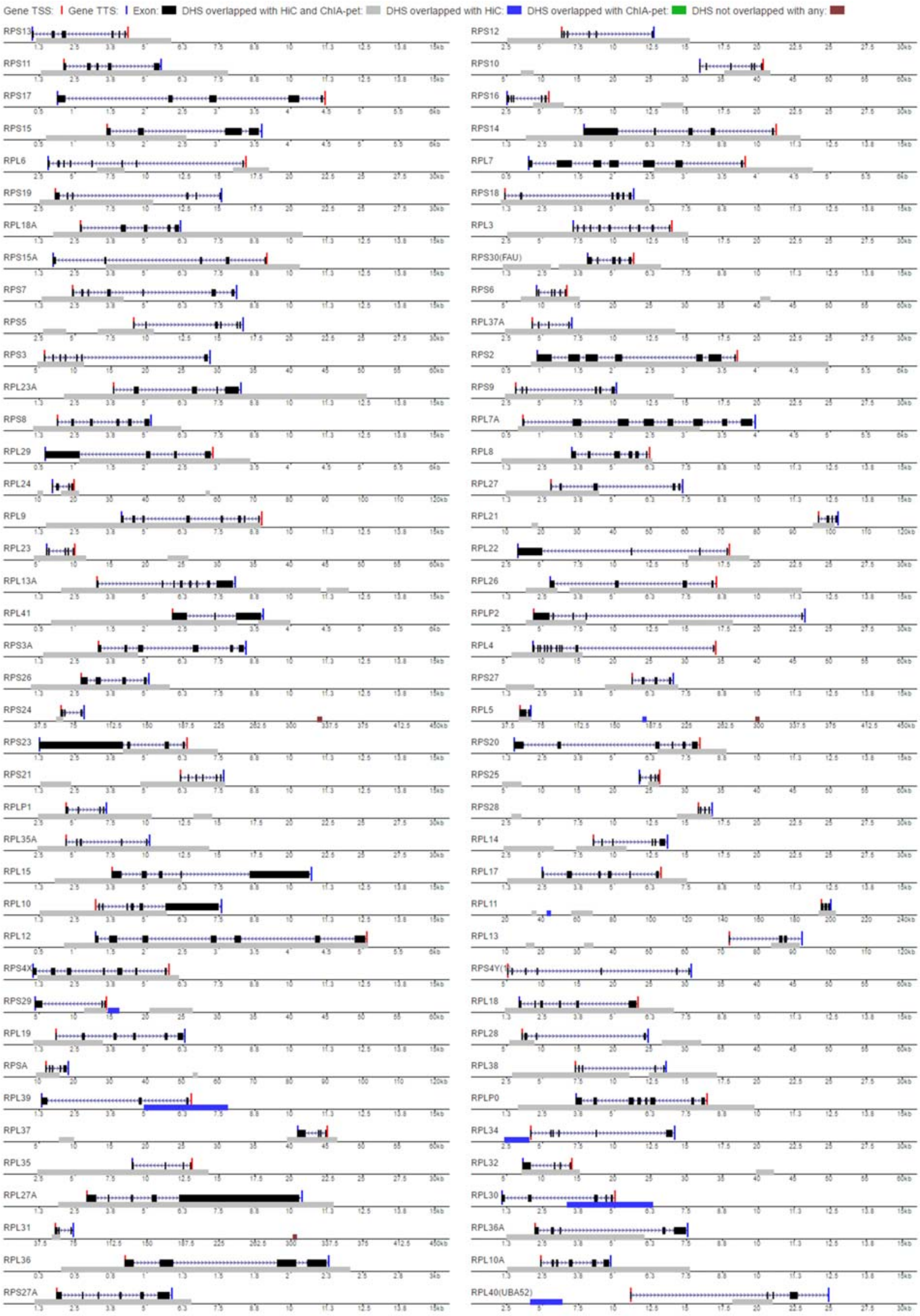

Figure S3. The predicted union regions. Each gene is shown in one row, followed by the ruler and the predicted regulatory regions.

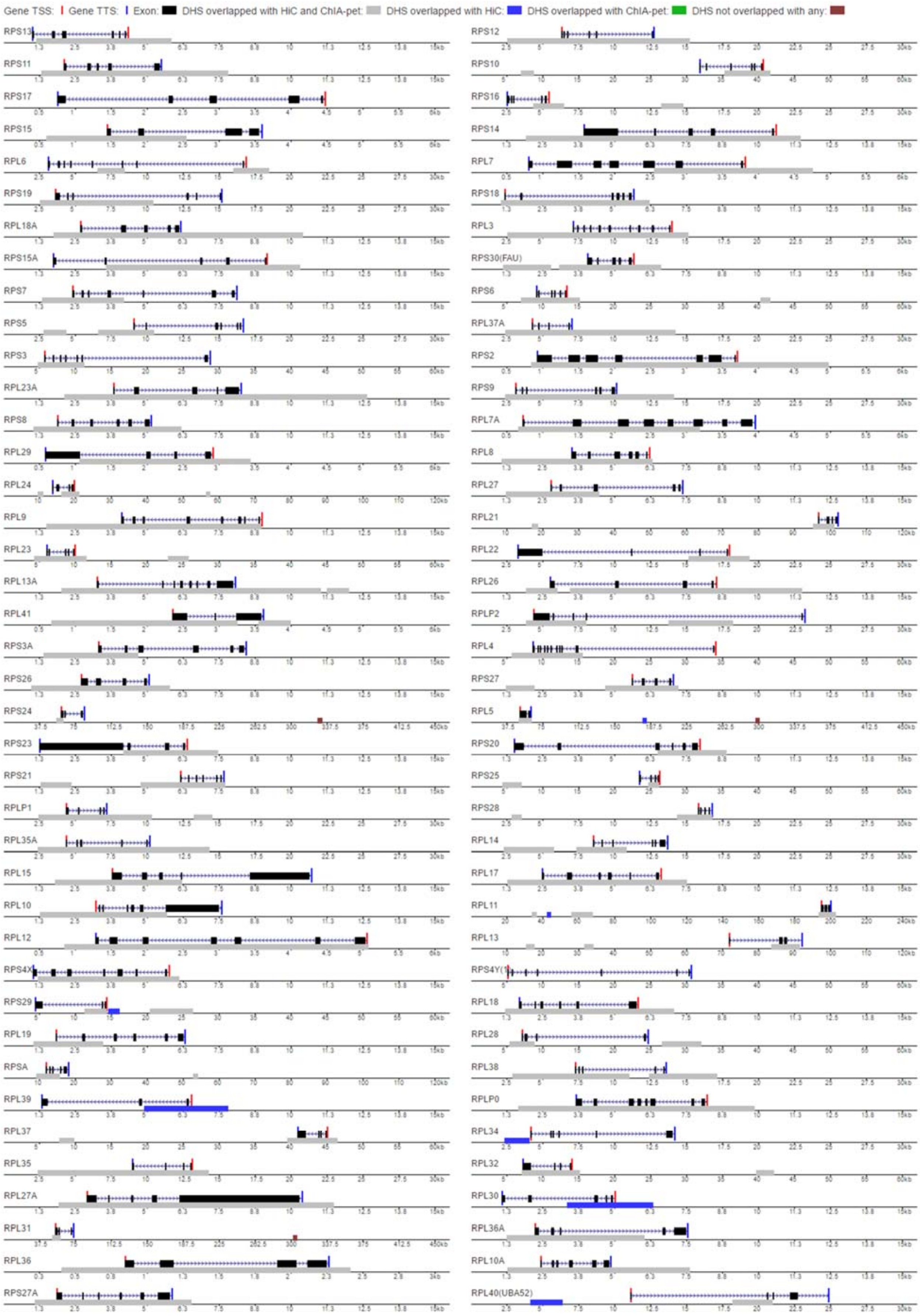

Figure S4. The predicted union 85% regions. Each gene is shown in one row, followed by the ruler and the predicted regulatory regions.
